# Survival Benefit of Traditional Chinese Herbal Medicine (A Herbal Formula for Invigorating Spleen) in Gastric Cancer Patients with Peritoneal Metastasis

**DOI:** 10.1155/2014/625493

**Published:** 2014-03-02

**Authors:** Lu Zhao, Ai-Guang Zhao, Gang Zhao, Yan Xu, Xiao-Hong Zhu, Ni-Da Cao, Jian Zheng, Jin-Kun Yang, Jian-Hua Xu

**Affiliations:** ^1^Department of Oncology, Longhua Hospital Shanghai University of Traditional Chinese Medicine, Shanghai 200032, China; ^2^Department of Oncology, Putuo District Central Hospital, Shanghai University of Traditional Chinese Medicine, Shanghai 200062, China; ^3^Department of Surgery, Renji Hospital Affiliated to Shanghai Jiao Tong University, Shanghai 200127, China

## Abstract

*Objective.* We evaluated the efficiency of traditional Chinese herbal medicine (a compound herbal formula for invigorating spleen) as a complementary and alternative therapy for gastric cancer patients with peritoneal metastasis. *Methods.* Between 2001 and 2012, 93 gastric cancer patients with peritoneal metastasis were enrolled in this study. The effect of traditional Chinese herbal medicine on their long-term outcome was investigated. Kaplan-Meier method was used to assess the difference in survival time, and Cox proportional hazards regression analysis was performed to identify independent prognostic factors. *Result.* First-line palliative chemotherapy plus traditional Chinese herbal medicine was performed in 47 patients and the other 46 patients received chemotherapy alone. The overall survival was different between patients with and without traditional Chinese herbal medicine (12.0 versus 10.5 months; *P* = 0.046). According to the Cox proportional hazard model, first-line chemotherapy cycle (hazards ratio [HR] = 0.527; 95% CI = 0.323~0.860) and TCHM (hazards ratio [HR] = 0.644; 95% CI = 0.481~0.992) were selected as independent prognostic factors for survival. *Conclusion.* The results suggest that traditional Chinese herbal medicine could improve the prognosis of the gastric cancer patients with peritoneal metastasis.

## 1. Introduction

Peritoneal metastasis is the most common form of recurrence and metastasis in gastric cancer patients. Because peritoneal metastasis has no special manifestation at early stage, once the patients have the serious complications, such as intestinal obstruction, obstructive jaundice, and malignant ascites, they will lead to difficult clinical treatment and poor prognosis. Current therapeutic methods for peritoneal metastasis of gastric cancer include cytoreductive surgery, intraperitoneal chemotherapy, systemic chemotherapy, and biotherapy; but the curative effects are generally unsatisfactory. Therefore, early diagnosis and effective comprehensive treatment is a key target for prolonging the survival time.

Comparatively speaking, traditional Chinese medicine (TCM) is more holistic in treating malignant tumor, whereas Western medicine is more direct and localized through the use of chemotherapy or radiotherapy. The traditional Chinese herbal medicine (TCHM) guided by the concept of holistic not only aims at local treatment, but also focuses on controlling the progression of tumor via adjusting systemic functional status. We designed this TCHM formula (a compound herbal formula for invigorating spleen, formerly named Wei Chang'An) to improve the survival of gastric cancer entirely based on TCM theory.

Our previous researchstudies indicated that this TCHM formula could induce gastric cancer cells apoptosis and suppress proliferation [1, 2]. Clinical study has indicated that this TCHM formula had a potential value in improving the prognosis of patients with advanced gastric cancer [3, 4]. Thus, the aim of the present study was to further investigate the effects of this formula on gastric cancer patients with peritoneal metastasis.

## 2. Materials and Methods

### 2.1. Patients Characteristics

Between April 2001 and April 2012, 93 gastric cancer patients with peritoneal metastasis who underwent chemotherapy plus TCHM or chemotherapy alone at the Department of Oncology, Longhua Hospital, Shanghai University of Traditional Chinese Medicine, and the Department of Surgery, Renji Hospital, Shanghai Jiao Tong University School of Medicine, were enrolled in this study. The patients' characteristics, pathological findings, and treating processes were collected retrospectively from individual patient records.

### 2.2. Pretreatment Examinations

The patients were regarded as having clinically evident peritoneal metastasis if they met one of the following clinical or imaging manifestations: (1) intraoperative pathological biopsy proves the dissemination of peritoneum, pouch of Douglas, and nodule, or the discovery of periumbilical metastatic nodules. (2) Positron emission tomography/computed tomography (PET/CT) [5]: 18F-FDG accumulation along peritoneum and intestinal wall and inside omental bursa; SUV max ≥ 2.5; the corresponding parts on CT images show morphological changes such as peritoneal thickening and nodules. (3) Magnetic resonance (MR) [6]: abnormally enhanced signals along the surface of mesentery, with unsmooth boundary. (4) Computed tomography (CT) [7]: massive ascites; omental changes (pie-like thickening, focal nodules, smudgy density shadow, and bursa-like changes in the omentum); remarkably increased fat density of the small or large bowel.

### 2.3. Treatment

All patients received first-line palliative chemotherapy depending on their pathological type, physical condition, and tolerance. The scheme was given for 5 to 8 days every 21 to 28 days, based on fluorouracil or together with platinum, anthracycline, or platinum.

The compound herbal formula aimed at invigorating the spleen. The major components were* Radix Pseudostellariae* (12 g), *Rhizoma Atractylodis Macrocephalae *(12 g), *Poria *(30 g), *Rhizome Pinelliae Preparata* (9 g), *green tangerine peel *(4.5 g), *Concha Ostreae *(30 g), and *Prunella vulgaris *(9 g).

The herbal components above were mainly provided by Sunbow Pharmaceutical (7600 Zhongchun Road, Shanghai 201100, China). This Chinese medicine factory has quality control (certified GMP ShanghaiG0172). The preparation of the formula water decoction and its quality control had been described in our previously published articles [2]. Each dose was 300 mL at the concentration of 40 g/L (*Radix Pseudostellariae*). One dose per day was taken 2–4 times, and the treatment continued for more than 3 months. According to patients' symptoms and syndrome differentiation, herbs were increased or reduced every 2 weeks.

### 2.4. Statistical Analysis

Kaplan-Meier method was used to assess the difference in survival time. The differences of the clinical and demographic factors were analyzed by log-rank test. In this study, overall survival (OS) time was defined as time from initial treatment (chemotherapy plus TCHM or chemotherapy alone) to the date of death from any cause.

The Cox proportional hazards regression analysis of survival includes sex, age, primary tumor location, type of histology, number of metastatic sites, first-line chemotherapy cycle, first-line chemotherapy scheme, and intraperitoneal chemotherapy. Differences were assumed to be significant when the *P* value was less than 0.05. All of the analyses were performed using the SPSS statistical software program package (SPSS version 16.0 for Windows).

## 3. Results

### 3.1. Patients

A total of 93 patients were included—47 received the chemotherapy plus TCHM and 46 underwent chemotherapy alone. None of the included patients ever received cytoreductive surgery.

In the patients with TCHM, the longest follow-up period was 111.09 months and the shortest was 3.95 months, 37 patients (78.7%) died of cancer, 6 patients (12.8%) were alive, and 4 patients (8.5%) lost followup. And in the patients without TCHM, the numbers were 43.46, 3.25, 44 (95.7%), 2 (4.3%), and 0 (0%), respectively.

The patients' characteristics and treatments provided are indicated in Table 1. And we compared the baseline between patients according to the treatment provided. There were no differences between any two groups.

### 3.2. Survival Analysis

**Figure 1:**
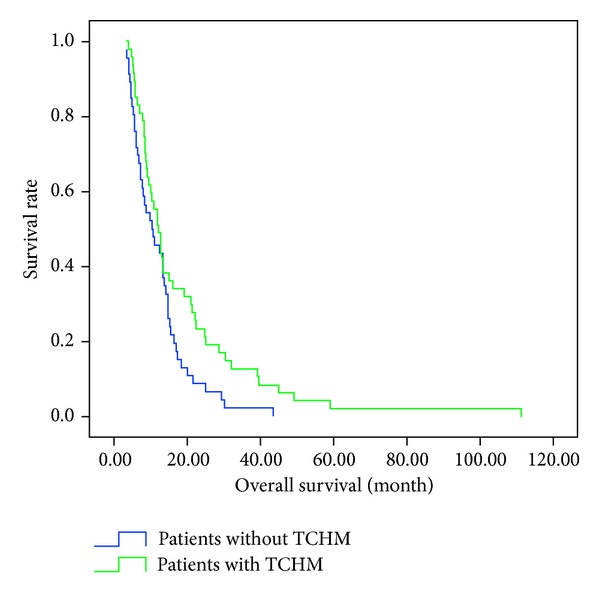
Survival curves of patients with and without TCHM. There is difference in MST between patients with TCHM (12.0 months) and those without TCHM (10.5 months; *P* = 0.026).

**Figure 2:**
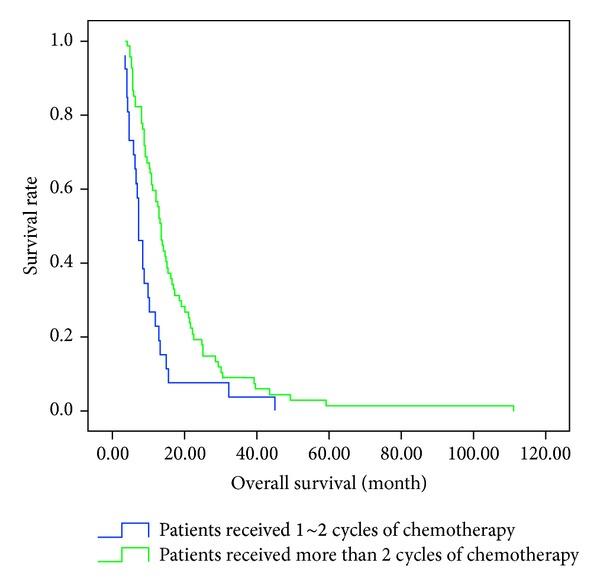
Survival curves of patients who receive 1~2 cycles and more than 2 cycles of chemotherapy. MST was significantly longer for patients who receive more than 2 cycles of chemotherapy (13.3 months) than those who received 1~2 cycles (7.0 months; *P* = 0.002).

Figure 1 shows the overall survival curves of patients with and without TCHM. The median survival time (MST) of patients with TCHM was 12.0 months and that without TCHM was 10.5 months (*P* = 0.026). Overall survival curves of patients who receive 1~2 cycles and more than 2 cycles of chemotherapy are shown in Figure 2. MST was significantly longer in patients who receive more than 2 cycles of chemotherapy (13.3 months) than in those who took 1~2 cycles (7.0 months, *P* = 0.002).

Multivariate analysis by Cox proportional hazard model showed that first-line chemotherapy cycle (*P* = 0.010) as well as TCHM (*P* = 0.460) were considered as independent prognostic factors for survival. The hazard ratio (HR = Exp[*β*]) of first-line chemotherapy cycle was 0.527, and 95% confidence interval was from 0.323 to 0.860. The HR of TCHM was 0.644, and 95% CI was from 0.418 to 0.992 (Table 2).

## 4. Discussion

Peritoneal metastasis is the predominant pattern among gastric cancer patients [8]. Diagnostic laparoscopy is considered the gold standard for diagnosis of peritoneal metastasis; but as a traumatic test, its clinical application is limited [9]. Peritoneal cytology has been used in the detection of subclinical peritoneal disease, and positive peritoneal cytology was defined as stage IV disease [10, 11]. PLC, however, has a low sensitivity and is not always positive in cases of visible peritoneal metastasis [12]. Currently, with the development of radiation immunoassay, enzyme-linked immunosorbent assay and RT-PCR for CEA mRNA, its low sensitivity, and high rate of missed diagnosis in detecting the intraperitoneal free cancer cells have been improved [13, 14]. Meanwhile, PLC is supplemented by the use of imaging techniques such as CT, MR, and PET/CT in the evaluation of peritoneal metastasis. As the diagnosis of peritoneal metastasis remains challenging, in this paper, we evaluated the peritoneal metastasis through focal pathology and imaging manifestations.

So far, there is no standard treatment for peritoneal metastasis. The 4th International Conference on Peritoneal Surface Oncology put forward cytoreductive surgery combined with perioperative intraperitoneal chemotherapy (IPC) for treatment of malignant tumors with peritoneal metastasis [15]. Cytoreductive surgery means that the limited peritoneal metastasis could be locally excised to reduce the tumor load maximally if the patient does not have liver or distant lymph node metastasis and IPC further kills out the micrometastasis and free tumor cells in the abdominal cavity left after surgery. A number of studies have shown that IPC has a positive function on prolonging the survival of advanced gastric cancer patients [16]. Recently, hyperthermia intraperitoneal chemotherapy (HIPEC) is widely applied in clinics, and studies have shown that cytoreductive surgery plus HIPEC were an independent prognostic factor on peritoneal metastasis from GC and recommended it as a standard treatment regimen for selected patients [17–21]. As few cases enrolled in the present study received intraperitoneal treatment (6 in the test group and 4 in the control group), the multifactor analysis did not indicate the IPC as an independent prognostic factor.

As a standard regimen for advanced or recurrent gastric cancer patients from the clinical studies, the efficiency of S-1 plus cisplatin for the patients with peritoneal metastasis remains to be seen [22]. Okabe et al. adopted the S-1 combined with cisplatin as induction chemotherapy on 41 gastric cancer patients with peritoneal metastasis. The result showed that, after chemotherapy, the survival benefit was significant in the patients with R0 resection compared to those with noncurative resection or without surgery, and it purposed that gastric cancer patients with limited peritoneal dissemination were sensitive to induction chemotherapy with S-1 plus cisplatin [23]. Due to its good transition from blood to the peritoneal cavity, paclitaxel was considered to be effective for peritoneal metastasis [24, 25]. In the OGSG0401 trail, the median survival time of gastric cancer patients with peritoneal metastasis who received treatment of paclitaxel (50 mg/m^2^ qd day 1 and 8) plus S-1 (40 mg/m^2^ bid day 1–14) scheme was 310 days, and the regimen demonstrated efficacy and tolerable toxicity [26]. The gastric cancer patients with peritoneal metastasis were excluded from clinical trials containing cisplatin for their complications such as ascites. MTX and 5-Fu sequential scheme was used based on the result from Japan Clinical Oncology Group (JCOG) 0106 trial for such patients [27]. Imazawa et al. reported that after treatment of methotrexate (MTX) combined with 5-Fu chemotherapy, the 31 gastric cancer patients with peritoneal metastasis had median OS of 255 days; of the 21 detectable focus patients, PR was observed in 4 cases; of the 26 patients complicated with ascites, the ascites amount decreased obviously in 14 cases [28]. Since effective regimen is under discussion to improve the poor prognosis of peritoneal metastasis from gastric cancer, systemic chemotherapy is the main treatment. Studies above showed that chemotherapy could improve the overall survival of gastric cancer patients with peritoneal metastasis. In our study, Cox proportional hazard model also showed that the chemotherapeutic cycle was an independent influence factor of prognosis. Nevertheless, the chemotherapy scheme was not indicated as an influence factor.

TCM developed from a totally different background from Western medicine. We designed this formula to improve the survival of gastric cancer based on TCM theory of the etiology and pathogenesis. In this compound formula, the herbs which have the function of invigorating the spleen function played the role of the sovereign ingredient. And the herbs for heat-clearing and detoxifying as well as for promoting circulation and removing blood stasis played the roles of minister and assistant ingredients. The major ingredients in this formula were *Radix Pseudostellariae*, *Rhizoma Atractylodis Macrocephalae*, *Poria*, *Rhizome Pinelliae Preparata*, *green tangerine peel*, *Concha Ostreae*, and *Prunella vulgaris*. And the attributes of the constituents of this compound formula are as follows: *Radix Pseudostellariae *and *Rhizoma Atractylodis Macrocephalae *are used to reinforce the spleen function supplement Qi (according to the fundamental theory of TCM, Qi is often translated as vital energy); *Poria *is used to invigorate the spleen and eliminate dampness; *Rhizome Pinelliae Preparata *is used to dry dampness and dissolve phlegm; *green tangerine peel *is used to promote the circulation of Qi and blood; *Concha Ostreae *and *Prunella vulgari *are used to soften hardness and dissolve lump.

In our previous studies, it was found that the TCHM (a herbal formula for invigorating the spleen) had certain antiperitoneal metastasis ability, a significant reduction in peritoneal metastasis lesions and ascites occurrence in the model of nude mice which were planted splenic-subcapsularly with human gastric cancer cell strain SGC-7901 [29]. And the TCHM could induce gastric cancer cell apoptosis and inhibit growth *in vivo*. Its mechanisms might be involved in the downregulation of Stat3, RIPX, ROD1, and Bcl-2 genes [1, 2].

Previous clinical paired comparative studies have indicated that advanced gastric cancer patients could benefit from TCHM. A clinical study of 399 patients with advanced gastric cancer suggested that patients who had received TCHM had better prognosis on multivariate analysis, independent of other prognostic factors (*P* = 0.000) [3]. In this present study, the OS of advanced gastric cancer with peritoneal metastasis could be prolonged by TCHM (12.0 versus 10.5 months; *P* = 0.046). Meanwhile, the multifactor analysis also showed that the TCHM has an independent prognostic factor (*P* = 0.460). The hazard ratio of TCHM was 0.644, and 95% CI was from 0.418 to 0.992.

Though there is limitation associated with retrospective study in providing robust evidence, the result of this study revealed the positive curative effectiveness of TCHM as a combination therapy for gastric cancer patients with peritoneal metastasis.

Based on our result, we proposed that TCHM aiming at invigorating the spleen combined with systematic chemotherapy could be useful for the treatment of gastric cancer with peritoneal metastasis. And with the development of cytoreductive surgery and intraperitoneal chemotherapy, there is still much room for the limited survival rates to improve.

*Safety and Toxicity*. The TCHM treatment was well tolerated. During the followup, there were no TCHM-related adverse events observed.

## 5. Conclusions

In this study, we showed that the traditional Chinese herbal medicine in combination with chemotherapy would improve the prognosis of gastric cancer patients with peritoneal metastasis than chemotherapy alone.

## Figures and Tables

**Table 1:** Patients and cancer baseline characteristics.

Characteristic	Treatment		*P* value
	TCHM (*n* = 47)	Non-TCHM (*n* = 46)	
Sex (%)			
Male	26 (55.3%)	34 (73.9%)	0.062
Age (%)			
<60	29 (61.7%)	33 (71.7%)	0.310
≥60	18 (38.3%)	13 (28.3%)	
Primary tumor site (%)			
Gastroesophageal junction	2 (4.3%)	2 (4.3%)	0.983
Stomach	45 (95.7%)	44 (95.7%)	
Histology (%)			
Intestinal	30 (63.8%)	31 (67.4%)	0.973
Diffuse	3 (6.4%)	1 (2.2%)	
Mixed	13 (27.7%)	12 (26.1%)	
Missing	1 (2.1%)	2 (4.3%)	
Number of metastatic sites (%)			
1~2	32 (68.1%)	31 (67.4%)	0.944
>2	15 (31.9%)	15 (32.6%)	
Chemotherapy			
1~2 cycles	10 (21.3%)	16 (34.8%)	0.150
3 cycles and more	37 (78.7%)	30 (65.2%)	
First-line chemotherapy scheme			
Single-agent regimen^a^	5 (10.6%)	2 (4.3%)	0.945
Joint regimen of 2 drugs^b^	17 (36.2%)	21 (45.7%)	
Joint regimen of 3 drugs^c^	21 (44.7%)	21 (45.7%)	
Other regimen^d^	4 (8.5%)	2 (4.3%)	
Intraperitoneal chemotherapy			
Nonintraperitoneal chemotherapy	40 (85.1%)	43 (93.5%)	0.197
Intraperitoneal chemotherapy	7 (14.9%)	3 (6.5%)	

^a^Fluorouracil.

^b^Fluorouracil plus cisplatin/oxaliplatin.

^c^Fluorouracil plus cisplatin/oxaliplatin plus anthracycline or paclitaxel/docetaxel.

^d^Fluorouracil plus others.

**Table 2:** Multivariate analysis of factors influencing survival of gastric cancer patients with peritoneal metastasis.

Factor	*B*	SE	Wald	Sig.	Exp(*β*)	95.0% CI for Exp(*β*)	
						Lower	Upper
Chemotherapy cycle	−0.640	0.250	6.570	0.010	0.527	0.323	0.860
TCHM	−0.440	0.221	3.977	0.046	0.644	0.418	0.992
